# Neutrophil extracellular traps mediate deep vein thrombosis: from mechanism to therapy

**DOI:** 10.3389/fimmu.2023.1198952

**Published:** 2023-08-23

**Authors:** Mengting Yao, Jiacheng Ma, Dongwen Wu, Chucun Fang, Zilong Wang, Tianting Guo, Jianwen Mo

**Affiliations:** ^1^ The First Clinical College, Gannan Medical University, Ganzhou, Jiangxi, China; ^2^ Department of Orthopedic Surgery, The First Affiliated Hospital of Gannan Medical University, Ganzhou, Jiangxi, China; ^3^ Department of Orthopedics, Guangdong Provincial People’s Hospital Ganzhou Hospital, Ganzhou Municipal Hospital, Ganzhou, Jiangxi, China

**Keywords:** deep venous thrombosis, neutrophil, neutrophil extracellular traps, platelet, deoxyribonuclease

## Abstract

Deep venous thrombosis (DVT) is a part of venous thromboembolism (VTE) that clinically manifests as swelling and pain in the lower limbs. The most serious clinical complication of DVT is pulmonary embolism (PE), which has a high mortality rate. To date, its underlying mechanisms are not fully understood, and patients usually present with clinical symptoms only after the formation of the thrombus. Thus, it is essential to understand the underlying mechanisms of deep vein thrombosis for an early diagnosis and treatment of DVT. In recent years, many studies have concluded that Neutrophil Extracellular Traps (NETs) are closely associated with DVT. These are released by neutrophils and, in addition to trapping pathogens, can mediate the formation of deep vein thrombi, thereby blocking blood vessels and leading to the development of disease. Therefore, this paper describes the occurrence and development of NETs and discusses the mechanism of action of NETs on deep vein thrombosis. It aims to provide a direction for improved diagnosis and treatment of deep vein thrombosis in the near future.

## 1 Introduction

Three major factors contribute to the formation of deep vein thrombosis: static blood flow, venous wall damage, and a hypercoagulable state (1). In isolated calf DVT, the plantar vein is most frequently involved (52.6%) (2), followed by peroneal vein thrombosis, gastrocnemius plexus, posterior tibial vein, and gastrocnemius vein (3). In the majority of cases, DVT is caused by the interaction of acquired risk factors (e.g., fracture, surgery, pregnancy, advanced age, inactivity, etc.) and genetic risk factors (e.g., gene mutation, etc.) (4, 5). Coronavirus disease 2019 (COVID-19) is an infectious disease that has broken out in recent years, and the incidence of DVT in patients with COVID-19 is 35.2% (6), becoming an important risk factor for DVT. In 2021, a report from the USA indicates that there are approximately 1,220,000 VTE patients in the USA each year (7). In the general population, the prevalence of DVT is estimated at 67 cases per 100,000 people per year (8). In patients with clinical symptoms, the prevalence of DVT is approximately 10-15% (9). DVT of the lower extremity affects 1% to 2% of hospitalized patients and is responsible for approximately 200,000 deaths per year in the United States (10). The incidence of DVT in the lower leg is higher in orthopedic surgery patients than in general surgery patients (11), with patients with femoral stem fractures (especially in the elderly) at risk of DVT from admission to surgery (12). Therefore, if DVT is not diagnosed and treated on time, it can affect the prognosis of the disease and the quality of life of the patient.

Neutrophils play a decisive role in the body’s intrinsic immune response and constitute the first line of defense against a wide range of pathogens. They eliminate invaders through phagocytosis, degranulation of antimicrobial proteins, synthesis of reactive oxygen species (ROS), and recruitment and activation of other immune cells. Neutrophils produce a specific series of responses to increasing numbers of stimuli, leading to chromatin decondensation and the subsequent formation of NETs. Activated neutrophils can activate and damage endothelial cells (13–15), and activated endothelial cells can trigger the release of NETs (16, 17), resulting in endothelial cytotoxicity and creating a vicious cycle of neutrophilic and endothelial cell activation that can lead to thrombosis. It has been found that a reduction in the number of neutrophils can lead to a substantial reduction in the size of the thrombus (18) and that neutrophils can influence TNF, NF-κB, and apoptotic pathways to play a role in thrombus formation (19).

NETs components principally comprise H3, histone H4, DNA, and other external proteins of origin, with DNA forming the NET backbone, providing scaffolding for various proteins. NET-associated neutrophil-derived proteins include myeloperoxidase (MPO), cathepsin G, neutrophil elastase (NE), and protease 3. NETs are decorated with various proteins and inflammatory mediators on their surface, representing a link between infection, inflammation, innate immunity, thrombosis, and cardiovascular disease (20). Non-neutrophilic proteins associated with NETs, such as tissue factor (TF), are major triggers of the coagulation cascade and facilitate thrombosis (21). NETs and neutrophil-derived proteins in the serum of COVID-19 patients are associated with a higher risk of thrombotic events (22, 23). Therefore, the purpose of this paper was to investigate the mechanism of NETs for deep vein thrombosis by describing their occurrence and development to act as a reference for the diagnosis and treatment of deep vein thrombosis in the future.

## 2 The occurrence of NETosis

When stimulated by pathogens such as viruses, bacteria, and fungi, neutrophils kill pathogens by phagocytosis and release of granulins and NETs in response to various infections and damage-associated molecular patterns (DAMPs) (24), which is an important process of innate immunity. In addition, autoantigens (25, 26), urate crystals (27, 28), low-density lipoprotein (oxLDL) (29, 30), cholesterol (31), high-mobility group box 1 protein (HMGB1) (32, 33), pro-inflammatory cytokines (e.g., IL-1β (34), IL-8 (35), and tumor necrosis factor-α (TNF-α) (36)) can also stimulate the formation of NETs, which in turn can cause the development of a variety of diseases. In an environment of high neutrophil density, stimulation with monosodium urate (MSU) crystals can induce the formation of aggregated NETs (aggNETs) (37). AggNETs can disrupt neutrophil recruitment and degrade cytokines and chemokines, thereby promoting disease regression and demonstrating anti-inflammatory potential (38–40). It is worth emphasizing that elevated granulocyte colony-stimulating factor (G-CSF) in cancer plasma triggers the release and metastasis of NETs and is an important role in cancer-associated thrombosis (41, 42). Besides, PMA, LPS, NO, the calcium carrier A23187, the potassium carrier nigericin, and Porphyromonas gingivalis (Pg) are commonly used as induction agents for NETs *in vitro* studies (43, 44). H. influenzae was found to induce the formation of NETs in COPD patients while activating IL-6 trans-signaling leading to an increase in soluble IL-6 receptors (45). This suggests that the formation of NETs can be stimulated both *in vivo* and *in vitro*, leading to the development of disease.

**Figure 1:**
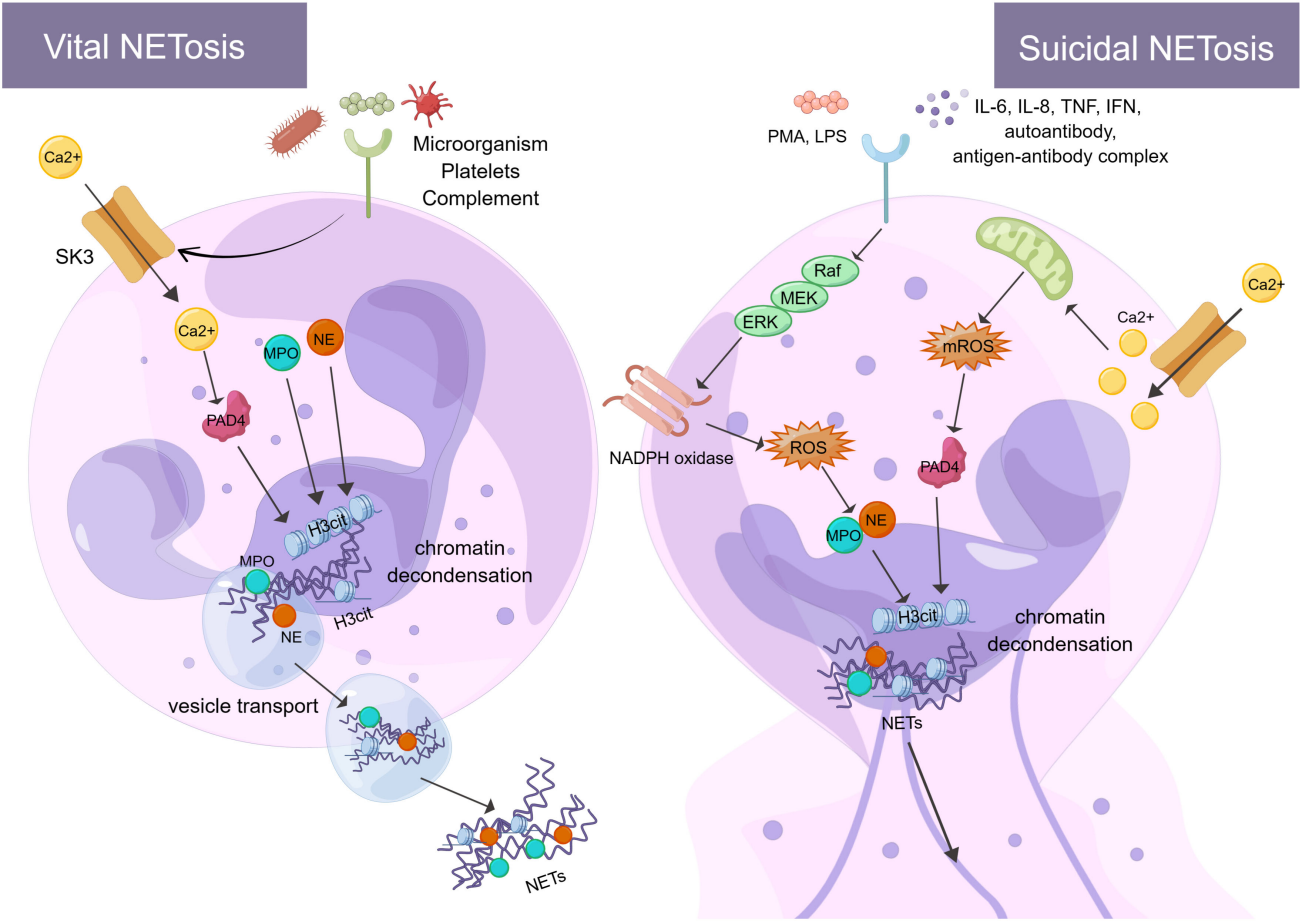
There are two processes of NETosis (By Figdraw). One is vital NETosis, which is activated by microorganisms, platelets, complement, etc. At the same time, Ca^2+^ is transferred to neutrophils via SK3 to activate PAD4, PAD4, NE, and MPO to induce histone citrullination in the nucleus. NETs are released from neutrophils when chromatin decondensation, nuclear membrane disassembly, and nuclear chromatin release occur. Finally, neutrophils release NETs into the extracellular space via vesicular transport. The other is suicidal NETosis, which is activated by *in vitro* stimuli (e.g., PMA and LPS) and *in vivo* stimuli (e.g., IL-6, IL-8, autoantibodies, TNF, IFN, and antibody-antigen complexes). These stimuli activate NADPH oxidase through the Raf-MEK-ERK signaling pathway, producing ROS. At the same time, ROS stimulates MPO causing activation of NE. In addition, Ca^2+^ transfer into neutrophils via SK3 stimulates mitochondria to produce mROS, which can activate PAD4. PAD4, NE, and MPO act together to cause histone citrullination and chromatin decondensation in the nucleus. Finally, neutrophils release NETs. The formation of NETs and their components play a crucial role in the immune and coagulation response. SK3, SK-channels; PAD4, protein-arginine deiminase type 4; NE, neutrophil elastase; MPO, myeloperoxidase; PMA, phor-bol-12-myristate-13-acetate; LPS, lipopolysaccharides; TNF, tumor necrosis factor; IFN, interferon; Raf, rapidly accelerated fibrosarcoma; MEK, mitogen-activated protein kinase; ERK, extracellular signal-regulated kinase; NADPH oxidase, nicotinamide adenine dinucleotide phosphate oxidase; ROS, reactive oxygen species; H3Cit, Histone H3 Citrullination.

The formation of NETs or the release of active NETs can be referred to as NETosis. There are two different forms of NETosis (**Figure 1**). The first is vital NETosis, which releases NETs after neutrophil migration, chemotaxis, and phagocytosis of pathogens. These occur independently of cell death and involve the expulsion of nuclear chromatin and the release of granule proteins. The other is suicidal NETosis, which refers to chromatin decondensation, nuclear membrane disassembly, and nuclear chromatin release (46). The main difference between the two types of NETosis is that suicidal NETosis implies cell death of neutrophils, whereas vital NETosis preserves living neutrophil functions (e.g., phagocytosis and chemotaxis) (47, 48). Vital NETosis is primarily caused by specific microbial molecules recognized by pattern recognition receptors (e.g., toll-like receptors). During this period, the neutrophils do not rupture. Neutrophils release DNA into the extracellular space via vesicular transport, a process that is very rapid (50-60 minutes) and not dependent on ROS and nicotinamide adenine dinucleotide phosphate (NADPH) oxidase (49). Small conductance calcium-activated potassium channels (SK-channels) are present on neutrophils and are the main calcium-activated potassium channels (50). After neutrophils are activated by microorganisms, platelets (51), and complement proteins, Ca^2+^ is transferred to neutrophils via SK-channels (52). Also, elevated intracellular calcium levels activate protein-arginine deiminase type 4 (PAD4), causing Histone H3 citrullination (H3Cit) and chromatin decondensation, which affects the formation of NETs (53).

Unlike vital NETosis, suicidal NETosis is dependent on PAD4 and NADPH oxidase in neutrophils (54) and lasts 2-4 hours (55). PAD4 catalyzes the citrullination/deimination of arginine residues of proteins, leading to the citrullination of histones, which is speculated to be critical for the formation of NETs. It has been found that reduced glutathione (GSH) activates PAD4 and that the concentration of GSH is comparable to that found in cells, thus requiring a reducing environment for PAD4 catalysis (56). More specifically, in suicidal NETosis, neutrophils can be stimulated *in vitro* (e.g., phorbol-12-myristate-13-acetate (PMA) and IL-8 (57)) and *in vivo* (e.g., autoantibodies, TNF, interferon (IFN) and antigen-antibody complex (25, 58, 59)) to activate NADPH oxidase via Raf-MEK-ERK (60). The NADPH oxidase complex on the neutrophil membrane produces ROS and delivers protein hydrolases to phagosomes to kill and remove pathogens (61, 62). This process mainly encompasses the activation and translocation of NE caused by ROS stimulation of MPO. NE translocates to the nucleus, degrades specific histones, and promotes chromatin decondensation (63). Neutrophil receptors such as dectin 1 inhibit the response of NETosis to small microorganisms by promoting phagosome formation and releasing NE from the nucleus (64). In addition to pathogens, excess fibronectin activates neutrophils via the Mac-1 integrin receptor, which triggers the production of ROS and NETs (65). NETs may also activate immune cells to target autoantigens, prompting interferon to trigger an inflammatory response and the formation of interferon immune complexes (66). It has been observed that patients with chronic sarcoidosis lacking NADPH oxidase were unable to form NETs. This validates the hypothesis that the formation of NETs is dependent on NADPH oxidase function and illustrates the significance of NET formation in human host defense (67). ROS production by the NADPH oxidase-dependent pathway is not the only source of ROS in neutrophils. Indeed, even in the absence of NADPH oxidase, mitochondrial ROS (mtROS) production can influence the formation of NETs (66). Interestingly, NETs contain mitochondrial DNA (mtDNA), but not nuclear DNA (68). This suggests that neutrophils can release mtDNA upon short-term activation, provided that they can produce mtROS and oxidize mtDNA (69, 70). Such mtDNA-rich NETs are mainly found in patients with chronic granulomatous disease (CGD) and systemic lupus erythematosus (SLE) (66). In addition, extracellular Ca^2+^ can cause the formation of NETs via SK-channels. This is also a non-dependent pathway of NADPH oxidase, which plays a specific role in the formation of NETs (71). The SK-channels can influence the production of mitochondrial ROS. For example, Panton-Valentine Leukocidin (PVL), a bicomponent leukotoxin released by Staphylococcus aureus (SA), induces the formation of NETs and mitochondrial oxidation with the involvement of SK-channels (72). In conclusion, although vital NETosis and suicidal NETosis play an important role in encapsulating, trapping, and killing pathogens, excessive release of NETs can trigger and amplify inflammatory responses, causing tissue damage and disease (e.g., vascular endothelial cell injury and thrombosis (73, 74)). Therefore, it is essential to limit excess NETosis and reduce damage to host organs.

## 3 Deep vein thrombosis and arterial thrombosis

Inflammatory and coagulation responses influence the formation of DVT, which includes the action of various molecules and cells. Following vascular injury, the collagen under the endothelium is exposed, and platelet adhesion to the collagen leads to platelet activation and release of endogenous adenosine diphosphate (ADP) and thromboxane A2 (TXA2), which in turn further recruit platelets for adhesion and ultimately a platelet hemostatic plug is formed to plug the wound and achieve hemostasis. In DVT, endothelial dysfunction is the trigger for platelet activation (75). Initially, in a flowing or static blood environment, platelets and red blood cells are the main components of the thrombus. After a few days, inflammatory cells (neutrophils, lymphocytes, and monocytes) infiltrate the margins of the thrombus, causing an inflammatory response. This process is dependent on endothelial activation and causes increased expression of cell adhesion molecules (e.g., p-selectin, e-selectin, and von Willebrand Factor (vWF) (76)), which promote the adhesion and activation of leukocytes. Vascular endothelial injury promotes the release of allergens and chemokines C3a and C5a from complement, and these can recruit and activate platelets, neutrophils, and monocytes. In a mouse model of venous thrombosis, the weight of the thrombin-antithrombin complex was closely correlated with C5a, suggesting that processes triggered during thrombosis promote C5a production (77). At the same time, increased platelet activating factor (PAF) and endothelin-1 cause vasoconstriction (78) and exacerbate inflammatory thrombosis. TF is known to trigger a clotting response in venous thrombosis. Inflammation exacerbates TF activation of exogenous coagulation pathways and the formation of extensive fibrin in venous vessels, where intravascular TF is seen mainly in monocytes and microparticles (79). This process is mainly mediated by the formation of a complex between TF and FVIIa, which activates FX. FXa is assembled into a thrombinogen complex that catalyzes the formation of thrombin. In the action of thrombin, fibrinogen is converted to fibrin (80). Inflammation also elevates the levels of phosphotidylserine, while decreasing thrombomodulin and inhibiting fibrinolysis (81). The above events exacerbate inflammation and thrombosis, where the presence of inflammation leads to damage to the peripheral venous wall and venous valves, resulting in valve dysfunction (82). After several weeks or more, the organized thrombus presents a fibrotic appearance with hemosiderin macrophages (83).

Neutrophils are the most abundant population of leukocytes in venous thrombosis. Although neutrophils may contribute to vein wall damage, they are sufficient to regulate fibrinolytic enzyme production and activity, promote fibrin and collagen lysis, and are essential for early thrombus lysis (84). The main treatment for DVT is thrombolysis, and plasmin is an important factor in early venous thrombolysis. D-dimer is a fibrin degradation product of fibrinolysis, which is clinically important for the diagnosis, efficacy assessment, and prognosis of thrombotic disease. Neutrophils can secrete matrix metalloproteases (MMPs), which activate the plasminogen activation system and attenuate the accumulation of inflammatory factors in DVT formation and venous thrombotic lesions (85). Tissue plasminogen activator (t-PA) induces the release of MMP-9 from neutrophils, a process that attenuates DVT formation through the NF-κB signaling pathway (86, 87). Meanwhile, MMP-2-dependent thrombolysis is an important compensatory mechanism for DVT regression, possibly representing a new treatment pathway (88). Macrophages produce various chemokines, inflammatory factors, and MMPs (e.g., uPA) that promote fibrinolysis and restore venous blood flow (85). However, when regulation reaches a certain limit, neutrophils can bind FXII and promote DVT formation through the release of NETs. Conversely, neutrophil reduction, FXII elimination, or breakdown of NETs can prevent DVT formation.

**Figure 2:**
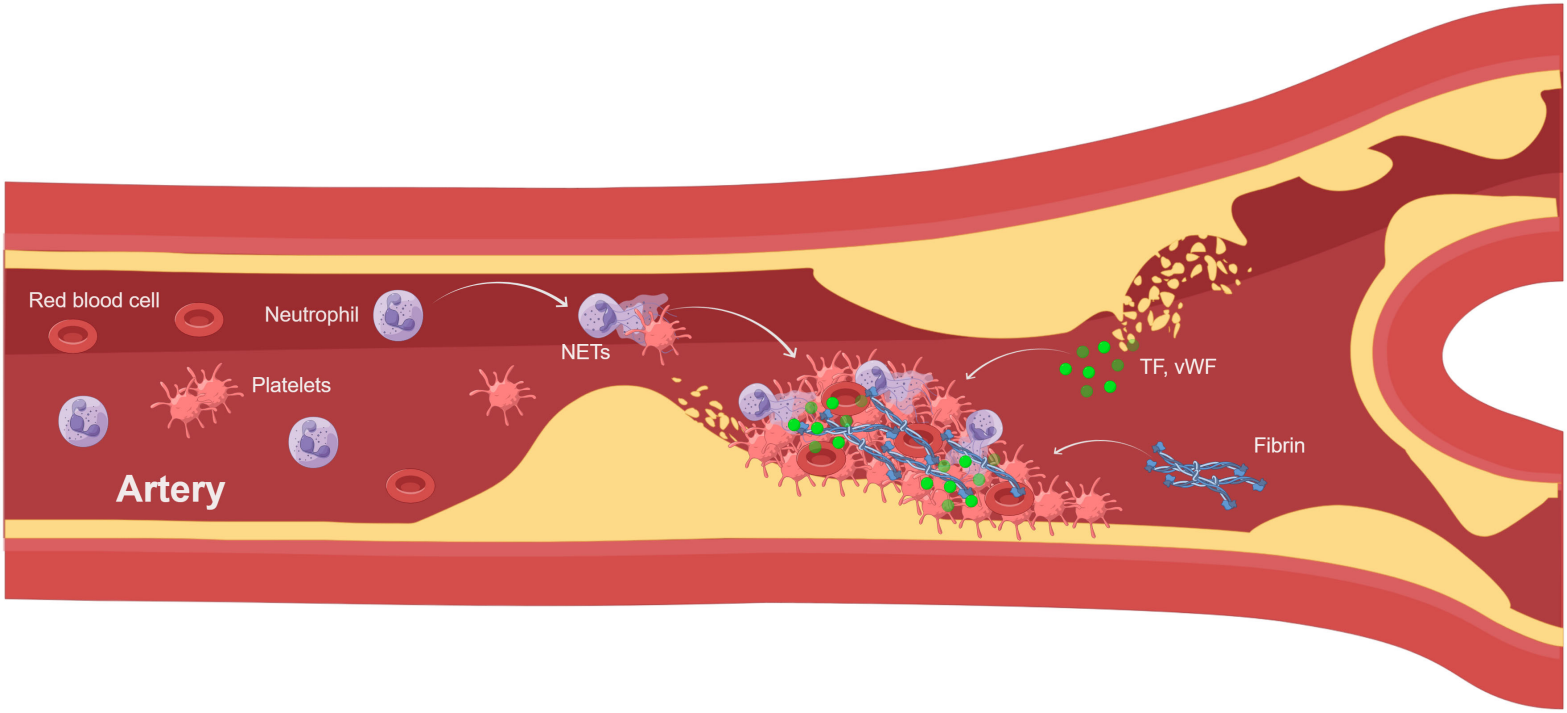
NETs involved in atherothrombosis (By Figdraw). Atherothrombosis is characterized by erosion or rupture of the atherosclerotic plaque, which exposes plaque material to the bloodstream and leads to arterial thrombosis. After plaque rupture, vWF and TF initiate extrinsic coagulation pathways. Platelet recruitment activates neutrophils leading to the formation of NETs.NETs enhance coagulation and inhibit fibrinolysis. The thrombus consists of NETs, platelets, and fibrin, leading to myocardial infarction and ischemic stroke. TF, tissue factor; vWF, von Willebrand factor.

Venous thrombosis and arterial thrombosis are two different types of thrombosis. Human venous thrombosis appears microscopically primarily as red areas rich in red blood cells and fibrin, and white areas composed primarily of platelets. However, arterial thrombosis generally begins after the rupture of an atherosclerotic plaque and has a white platelet-rich appearance, which is also common in acute events caused by the dislodgment of a thrombus. Atherothrombosis is mainly caused by chronic inflammation and acute lesions, such as atherosclerotic plaque rupture, acute myocardial infarction, and ischemic stroke. The degree of infiltration of neutrophils, one of the drivers of atherosclerosis, is related to the pro-inflammatory state and plaque stability (89). By analyzing human atherosclerotic plaques, scholars have found that intact plaques do not contain NETs, whereas adjacent vascular tissues of non-intact plaques contain large numbers of neutrophils and NETs. Meanwhile, neutrophils and NETs were confined to all types of complex lesions, with no significant differences in rupture, vesiculation, intraplaque hemorrhage, and thrombosis (90, 91). Thus, NETs are important players in atherosclerotic thrombosis, influencing plaque stability and the progression of disease complications (**Figure 2**). In addition to atherosclerosis itself, NETs released by neutrophils induce smooth muscle cell death, leading to reduced plaque stability and triggering acute myocardial infarction (92). NETs were found in arterial thrombi from acute myocardial infarction in mice and humans (93–95), and most of the arterial thrombi were positive for H3Cit (96), suggesting that neutrophils in arterial thrombi are in a highly activated state. Platelets were found in the vicinity of NETs, suggesting that activated platelets actively promote NETs in coronary thrombi (97). NETs burden is significantly higher in coronary thrombosis compared to venous thrombosis and is positively correlated with infarct size (98). Meanwhile, activation of platelets and neutrophils increases the risk of major adverse cardiovascular events after acute myocardial infarction (99). In addition, NETs enhance fibroblast activation and differentiation to promote myocardial fibrosis (100), affecting vascular health and exacerbating the thrombotic inflammatory response. Similarly, NETs have been found in thrombi from patients with ischemic stroke, with NETs being abundant in cardiac stroke thrombi (101) and associated with severity and mortality. Elevated H3Cit levels in ischemic stroke patients were positively correlated with extensive white matter lesion, which once again demonstrated the important role of NETs in the onset and progression of ischemic stroke (102). Meanwhile, NETs may promote t-PA resistance in acute ischemic stroke by activating platelets and endothelial cells. Therefore, strategies targeting NETs may be a therapeutic approach to improve the efficiency of t-PA thrombolysis in patients (103). In conclusion, activation of neutrophils and formation of NETs play a crucial role in venous thrombosis and arterial thrombosis, affecting disease progression and prognosis.

## 4 NETs mediate DVT formation

**Figure 3:**
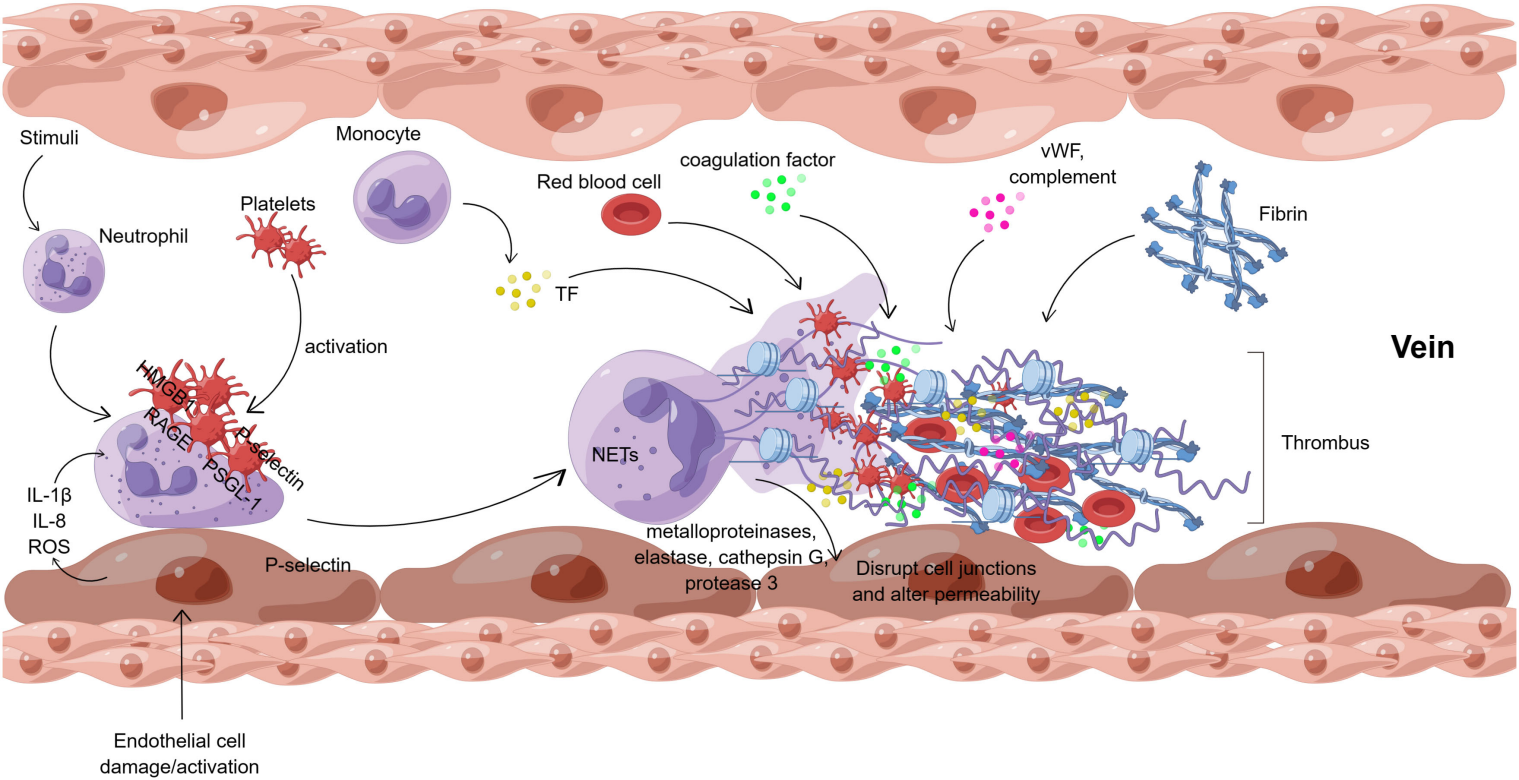
Interaction of NETs with cells and molecules in venous thrombosis (By Figdraw). After endothelial cell injury or activation, endothelial cells directly regulate neutrophils by releasing cytokines (IL-1β, IL-8, ROS, etc.). Meanwhile, *in vivo* and *in vitro* stimuli can also activate neutrophils directly. Besides, platelets can activate neutrophils, mainly mediated by HMGB1/RAGE and P-selectin/PSGL-1 interactions. Neutrophil activation results in the formation of NETs, which release metalloproteinases, elastase, cathepsin G, and protease 3 to disrupt the junctions between endothelial cells and alter the permeability of the vascular endothelium. NETs provide a scaffold for platelets, vWF, and fibrin, which together with TF, coagulation factors, complement, red blood cells, etc., eventually lead to venous thrombosis. ROS, reactive oxygen species; HMGB1, high mobility group protein B1; RAGE, receptor of advanced glycation end; PSGL-1, P-selectin glycoprotein ligand -1; vWF, von Willebrand factor; TF, tissue factor.

Neutrophil extracellular traps (NETs), which are composed of DNA and various proteins, influence DVT formation through several pathways. **Figure 3** shows the interaction of NETs with cells and molecules in venous thrombosis.

### 4.1 Endotheliocyte and NETs

NETs and endothelial cells play a synergistic role in thrombosis. Endothelial cells directly modulate neutrophil activity through the release of cytokines such as IL-1β, IL-8, and ROS, thereby accelerating the formation of NETs and regulating platelet function (104, 105). Meanwhile, NETs activate endothelial cells, promote the production of TXA2 and P-selectin by platelets, activate vascular cell adhesion molecule 1 (VCAM-1) and intracellular adhesion molecule 1 (ICAM-1), facilitate neutrophil migration, and enhance neutrophil-endothelial cell interactions (104, 106). One study found that this may be associated with the Gab2 protein (Grb-associated binder to adaptor signaling protein) pathway (107). Activated endothelial cells can influence thrombin production, enhance blood clotting and inhibit anticoagulation, thereby promoting thrombosis (108, 109). Endothelial cell activation is present in patients with superficial vein thrombosis and spontaneous venous thromboembolism (110, 111). Additionally, new research findings suggest that complements and neutrophils can lead to adverse immune responses that can aggravate inflammation and thrombotic disease (112). Furthermore, NETs can activate the complement system, exacerbate endothelial damage and promote platelet activation. Meanwhile, complement factor C3 deficiency attenuates arterial thrombosis in mice due to the disruption of the C3a-C3a receptor axis in platelets (113). In summary, endothelial cells can induce the release of NETs and activated neutrophils can cause endothelial activation and injury, which creates a vicious cycle between neutrophils and endothelial cells, leading to thrombosis.

### 4.2 Interaction between NETs and platelets

Platelets have hemostatic, immune, and anti-inflammatory functions and survive in circulation for about 7 days. A previous study investigated the effect of NETs produced *in vitro* on washed platelets and described that NETs produced *in vitro* directly induced platelet aggregation and provided a scaffold for platelet aggregation (114). The platelet surface purinergic receptor type Y, subtype 12 (P2RY_12_), binds to APD and plays an instrumental role in early DVT formation (115, 116). NETs not only provide a scaffold for platelet adhesion and aggregation but may also interact with fibrin to influence thrombus stability (117). The presence of vWF and platelets in areas of positive H3Cit staining in DVT was observed by assessing venous thromboembolism in surgical or autopsy specimens, demonstrating not only that histone citrullination occurs in DVT, but also that NETs, vWF, and platelets influence the formation and stability of DVT (83). Platelet dense granules contain inorganic polyphosphate (polyP), a key component associated with procoagulant activity (118). It is an intensely anionic polymer that promotes coagulation and NETs formation which is closely associated with thrombosis (119). In short, NETs interaction with platelets affects thrombus formation and stability.

#### 4.2.1 Relevant receptors mechanism

As mentioned, in the background of inflammatory thrombosis, platelets are activators of neutrophils. This process is dominated by the binding of P-selectin on activated platelets to P-selectin glycoprotein ligand -1 (PSGL-1) on neutrophils, as well as by the interaction of HMGB1 with the receptor of advanced glycation end (RAGE) (120). This subsequently causes Ca^2+^ translocation, ROS production, and activation of cellular pathways. Nuclear translocation of NE and MPO drives chromatin decondensation, NETs are released extracellularly, and platelets and NETs together promote coagulation and thrombosis. P-selectin/PSGL-1, HMGB1/RAGE, and GPIbα/Mac-1 play crucial roles in this process.

P-Selectin is expressed in platelets and stored in alpha granules. Upon platelet activation, it is translocated to the platelet surface where it binds to PSGL-1. PSGL-1 induces neutrophil activation through downstream signaling by tyrosine kinases and integrin activation. Simultaneously, PSGL-1 induces integrin activation on platelets in a p-selectin-dependent manner and increases platelet aggregation driving thrombosis (121). Studies in inflammatory mice have shown that p -selectin is important for the initial rolling of neutrophils in the vessel wall and is required for neutrophil recruitment to sites of inflammation or infection (122). Furthermore, in a mouse model, P-selectin in neutrophils activated the synergistic effects of PSGL-1 and C-X-C motif chemokine receptor 2 (CXCR2), thereby promoting neutrophil adhesion in blood flow-restricted veins, stimulating the release of NETs, and ultimately leading to deep vein thrombosis (123, 124). P-selectin promotes NETs formation by binding to PSGL-1, suggesting that this pathway is a potential therapeutic target for NETs-related diseases. P-selectin/PSGL-1 inhibitors are currently in clinical development as therapeutic agents to reduce inflammation and pathologic thrombosis (123). In summary, activation of P-selectin on platelets is necessary for platelet-mediated induction of NETs.

After signaling is established through P-selectin/PSGL-1, other co-stimulatory signals between platelets and neutrophils can also promote NETs formation. NETs induce platelets to enter a hypercoagulable state through the upregulation of cellular phosphatidylserine (PS) and P-selectin expression levels (125). This process is mediated by platelet HMGB1 (123, 126) binding to the receptors (RAGE, TLR2, and TLR4) on neutrophils. It was discovered that HMGB1 promotes neutrophil activation through the TLR4/NADPH pathway, which is dependent on NADPH oxidase (127). Subsequently, platelet activation via the TLR4-MyD88 pathway promotes thrombotic vessel obstruction (128, 129), signaling that HMGB1 is also a trigger for the formation of NETs (130).

Glycoprotein Ibα (GPIbα) is a platelet receptor that interacts with neutrophil integrin Mac-1 (αMβ2, CD11b/CD18). The binding of GPIbα to Mac-1 stabilizes platelet-neutrophil and is critical for the development and progression of thrombotic inflammatory diseases (131). At the same time, platelet-derived protein disulfide isomerase (PDI) promotes platelet-neutrophil interactions under conditions of thrombotic inflammation by regulating the function of GPIbα. In a mouse model of stroke, PDI-GPIbα signaling was found to play a crucial role in tissue injury and thrombosis (132). It was found that targeting GPIbα led to a significant reduction (44%) in platelet-mediated neutrophil invasion in brain inflammation. These results suggest that therapeutic blockade of platelet GPIbα could limit the deleterious effects of excessive inflammation while minimizing bleeding in the brain due to thrombocytopenia (133). For example, CCP-224, a specific GPIbα inhibitor, attenuates platelet-neutrophil interactions in the blood of patients with Sickle cell disease (134). In addition, Mac-1 deficiency or mutations in the Mac-1 binding site of GPIbα delay thrombosis after injury to large and small arteries (135). Meanwhile, Mac-1 may be involved in neutrophil cytoskeletal changes and the release of NETs (136). In summary, selective inhibition targeting GPIbα and Mac-1 may be an effective therapeutic approach to attenuate thrombotic inflammatory diseases.

#### 4.2.2 Platelets and neutrophils

Platelets and neutrophils are synergistically involved in the inflammatory response and thrombogenic processes, which provide a new connection between inflammation and thrombosis. The molecular mechanism of interaction between the two cells is mainly the binding of p-selectin on activated platelets and PSGL-1 on neutrophils, known as platelet-neutrophil aggregates (PNAs). Animal models have shown that PNAs are responsible for neutrophil recruitment in various cardiovascular and inflammatory diseases. Platelets promote neutrophil adhesion to the vascular endothelium through the upregulation of integrins and enhanced responsiveness to chemokines (137). Activated platelets, inflammatory stimuli, and chemical compounds can induce the formation of neutrophil-activated NETs (138, 139). In addition, platelet TLR4 activation also promotes the formation of PNAs and the generation of NETs (51). Some clinical trials have indicated that elevated levels of PNAs are associated with a higher risk of DVT, which affects the coagulation and immune response *in vivo* (140). Neutrophils create a specific fluid environment in inflamed microvessels that effectively promotes platelet aggregation to form thrombi (141). Besides, P2RY_12_ inhibitors such as clopidogrel can inhibit platelet-neutrophil interactions (142). It further indicates that disrupting a specific link between the immune and coagulation systems may be conducive to the prevention and treatment of microvascular thrombotic lesions. In conclusion, a better understanding of platelet-neutrophil interactions and their role in venous thrombosis would be beneficial in the prevention and treatment of DVT.

### 4.3 NETs and coagulation

NETs bind to some coagulation factors to trigger the clotting reaction and promote thrombosis. Coagulation factors (F) XII and FXI play crucial roles within the coagulation pathway. Autoactivation of FXII occurs when it’s exposed to a negatively charged surface, while FXIIa catalyzes the activation of FXI, which in turn activates FIX. FXII and FXI have been shown to contribute to thrombosis in animal models. Following exposure to pathogens, NETs bind to FXII and stimulate fibrin formation and thrombus stabilization (143). In DVT, NETs are essential for the autoactivation of FXII, coagulation factor assembly, and fibrin formation (144). DNA and histones have been reported to activate FXII and increase TF-dependent thrombin production. Meanwhile, FXII binds with the urokinase plasminogen activator receptor to promote neutrophil adhesion, chemotaxis, and NETs formation (145). In venous thrombosis models, FXII and FXI contribute to thrombus formation, which is correlated with elevated levels of citrullinated histone H3 (146). This implies that NETs may regulate levels of FXII and FXI, causing venous thrombosis. Thus, the interaction between NETs and some coagulation factors can promote thrombosis.

Thrombin cleaves fibrinogen into fibrin, which is the rate-limiting step in the coagulation cascade reaction. NETs promote the formation of peripheral blood fibrin and thrombin through the intrinsic pathway of coagulation (103). NE and MPO in NETs oxidize tissue factor pathway inhibitor (TFPI) and thrombomodulin, and inactivation of these endogenous anticoagulants may promote coagulation (147). Components of NETs, such as histones H3 and H4, are highly cytotoxic to endothelial cells (148) and smooth muscle cells (92) and induce platelet aggregation via Toll-like receptors 2 and 4 (117, 148), ultimately promoting thrombin production (117). Histones further contribute to thrombin production by binding to coagulation regulatory proteins and preventing the activation of activated protein C (APC) (149). Recent data also point out that neutrophils can be directly activated by heparin/platelet factor 4/IgG antibody complexes through their FcγRIIa receptors, which trigger the formation of NETs and thrombi (150). DNA, histones, and neutrophil extracellular traps exert anti-fibrinolytic effects in a plasma environment (151). The addition of DNA or histones to clotted plasma leads to fibrin thickening. In contrast, the addition of NETs to plasma clots significantly delays clot lysis *in vitro* by down-regulating tPA activity (152, 153). In a baboon DVT model, inhibition of neutrophil infiltration caused unstable clot formation, suggesting a role for neutrophils and NETs in clot stabilization (154). It is crucial to understand fibrin structure and properties through DNA and histones for fibrinolysis, thrombus stabilization, and lysis.

### 4.4 Infection, NETs and DVT

It is well known that there is an inextricable link between infections, NETs, and DVT. Microorganisms such as bacteria, viruses, and fungi can enhance the body’s immune defense against microorganisms by inducing the release of NETs from neutrophils through the activation of neutrophil receptors (TLR and NLR, etc.) and cytokines. Endothelial cells have natural anticoagulants such as glycosaminoglycans (GAGs), including heparan sulfate, dermatan sulfate, and heparin (155). In addition, endothelial cells also promote leukocyte recruitment and adhesion, and the expression of signaling molecules to actively fight inflammation. Following an inflammatory response to infection, endothelial cells lose their anti-coagulant and anti-inflammatory properties and initiate repair to eliminate the damage. The release of metalloproteinases, elastase, cathepsin G, and protease 3 by NETs has proteolytic activity, which can disrupt the junctions between endothelial cells (156) and alter the permeability of vascular endothelium. Endothelial dysfunction is associated with the activation of NET-externalized MMPs, and inhibition of MMP-2 activation restores endothelium-dependent function and reduces NETs-induced vascular cytotoxicity (157). Further activation of MMP amplifies hyperpermeable signaling, mainly by disrupting cytokines and chemokines (e.g., IL-1β, TNF-α, and CXCL8) (158, 159). In addition, in a mouse model of LPS-induced acute lung injury, histones in NETs may lead to host cytotoxicity and may be involved in lung tissue destruction (160). It has been demonstrated that NETs can induce pro-inflammatory and pro-angiogenic responses in human pulmonary artery endothelial cells (HPAEC) through MPO/H_2_O_2_-dependent activation of TLR4/NF-κB signaling (161). Excessive release of neutrophil-derived ROS can directly attack adherens junctions, tight junctions, and actin filaments, leading to endothelial barrier disruption (162). Neutrophil-produced ROS promotes endothelial dysfunction through the oxidation of key cellular signaling proteins such as tyrosine phosphatases. ROS also regulates intracellular free Ca^2+^ concentration, activates Ca^2+^/calmodulin-dependent myosin light chain kinase (MLCK) and promotes cytoskeletal reorganization, which induces changes in endothelial cell shape and increases vascular permeability (163). Therefore, the structural and functional integrity of the endothelium is essential for maintaining vascular homeostasis and preventing deep vein thrombosis.

In addition to causing endothelial dysfunction, the infection affects coagulation, leading to slowed or even stagnant venous blood flow, increasing the risk of thrombosis. It has been reported that inflammatory factors and chemokines (IL-1, IL-6, IL-8, and TNF-α) are elevated in COVID-19 patients (164). IL-6 is a crucial cytokine that is notably increased in COVID-19-infected patients. IL-6 induces TF expression, promotes fibrinogen, factor VIII, and platelet production, and is an essential activator of coagulation disorders (165–167). Patients can have a five-fold increase in fibrinogen levels, and this high fibrinogen level drives high blood viscosity, which, combined with low antithrombin levels, results in a high risk of venous and arterial thrombosis. A study found that circulating NETs were significantly elevated in patients with COVID-19 combined with thrombosis, and there was a strong relevance between NETs and D-dimer (fibrin degradation products). Although patients were treated with prophylactic anticoagulation, residual NETs in the circulation continued to increase the risk of thrombosis (22). Therefore, we believe that NETs are essential in COVID-19-associated thrombosis. In infection-induced stenosis and stagnation, NETs can enhance the viscosity and strength of blood clots, thereby increasing clot stability. NETs can increase the viscosity and strength of blood clots, thus increasing the stability of the clot. Due to the increased viscosity of the thrombus, it is more difficult for blood to pass through the blood vessels, which may result in slower blood flow. The DNA released during the formation of NETs can affect the flow of blood (168). At the same time, particle velocity experiments have shown that NETs associated with the vascular surface can slow blood flow and induce stasis even in the absence of other coagulation factors (169). Because animals do not develop spontaneous DVT, animal models of DVT play an important role in understanding the mechanisms of DVT formation (170). In the inferior vena cava stenosis model of DVT, NETs were found to be an integral presence in the formation of DVT (126). Following induction of blood flow restriction, NETs biomarkers accumulate in plasma over several hours and increased histones lead to elevated plasma vWF levels (171). Interestingly, NE was not required for the production of neutrophil NETs in mice *in vitro* under non-infectious stimuli.NE deficiency had no significant effect on thrombosis in the venous stenosis model (172). The stenosis model of DVT induces thrombus continuous exposure to the surrounding blood flow, whereas the stasis model of DVT does not. Thus, in a static environment, NETs have reduced contact with exogenous enzymes, which would release more histones and promote thrombosis directly. In the stasis model, neutrophils lacking TLR9 release more NETs, which contribute to venous thrombosis formation (173). At the same time, the absence of TLR9 signaling increases the formation of NETs, suggesting that TLR9 signaling is important for NETs clearance (174). Some scholars applied microfluidic techniques to study the influence of hemodynamics on NETosis during sterile thrombosis, and they found that high interstitial hemodynamics triggered rapid NETs release (175). Thus, the effect of blood flow velocity on NETs in deep vein thrombosis is complex and may vary depending on different factors.

The infection affects venous blood flow causing slow and stagnant blood flow while causing hypoxemia in some organs. Hypoxia increases the production of NETs, causing recurrent and worsening disease, which exacerbates inflammation and thrombotic disease. In COVID-19, infection activates inflammatory and coagulant responses, causing reduced lung perfusion. Deficient ventilation-perfusion triggers asymptomatic hypoxemia (176, 177). In addition, neutrophil infiltration of hypoxic tissues is characteristic of numerous acute and chronic infectious and inflammatory diseases. Hypoxia enhances the neutrophil degranulation response and may increase the production of NETs (178). In acute respiratory distress syndrome, excessive release of NETs causes alveolar dilatation, leading to extensive lung injury and hypoxemia (179). Hypoxia regulates NETs formation, deepening our understanding of the impact on neutrophil biology during the onset and resolution of inflammation. At the same time, hypoxemia is related to the risk of thrombosis and hypoxia can accelerate thromboembolic events *in vivo*. Hypoxia-inducible factor 1-alpha (HIF-1α) is an important regulatory protein in the hypoxic environment that mediates Nod-like receptor protein-3 (NLRP3) expression and increased IL-1β secretion and plays a vital role in the process of venous thrombosis (180). The ability of HIF-1α to regulate NLRP3 expression links the hypoxic response to the inflammatory state, suggesting that hypoxia can regulate important pathological effects through its ability to promote the inflammatory response. HIF1 has been found to down-regulate the expression of protein S (PS) (a natural anticoagulant that inhibits the coagulation factor IXa) in the liver, and decreased PS levels are associated with increased thrombin and increased thrombosis (139). In conclusion, the regulation of thrombogenesis and antithrombotic genes by hypoxia is currently under investigation, and other hypoxia-controlled mechanisms of thrombosis are expected to be discovered.

### 4.5 NETs and DVT complications

DVT complications include Pulmonary embolism (PE) (6%-32%) and post-thrombotic syndrome (PTS) (25%-38%). PE is a common acute complication of DVT with a mortality rate of 5%-10% (181). The dislodged clot can travel down the circulation into the pulmonary artery and cause massive PE, which can easily lead to patient death. Studies have shown that plasma fibrin clot is enhanced with NETs formation in patients with severe acute PE and that circulating H3Cit in acute PE correlates with disease severity (182). If the clot does not lyse in time in patients with PE, it may develop into chronic thromboembolic pulmonary hypertension (CTEPH). CTEPH is a chronic obstructive pulmonary artery disease. Studies have shown that NETs are an upstream trigger of TGF-β-mediated thrombofibrosis (183). We have previously learned that the release of NETs enhances thrombus stability (83), but based on current studies we do not know whether thrombus shedding is associated with NETs. In addition to this, PTS is the most common and important complication. The main clinical manifestations are painful edema, varicose veins, and hyperpigmentation. In severe cases, local ulceration of the lower limbs, which affects the patient’s quality of life. One study found that patients with DVT with PTS had higher levels of activated neutrophil-secreting nucleosomes and more pronounced apoptosis than patients with DVT without PTS (184). In addition to their pro-fibrinolytic, anticoagulant, and anti-platelet effects, statins may reduce neutrophil and NETs levels and exert anti-inflammatory effects. Therefore, statins may be used to improve DVT and reduce PTS (185). NETs are associated with the severity of DVT complications, and the function and prognostic value of NETs in DVT require further exploration.

### 4.6 Other molecules involved in DVT formation

Other molecular mechanisms affecting the formation of NETs and DVT are listed below. Slc44a2 is a transmembrane neutrophil surface protein that acts as a receptor for vWF. Expression of human neutrophil antigen 3b (HNA-3b) epitopes correlates with the risk of human venous thrombosis (VT) (186). Activated platelet integrin αIIbβ3 can interact with Slc44a2 to bind neutrophils directly (187). It has been found that Slc44a2 is involved in the direct adhesion and activation of neutrophils to vWF and that Slc44a2/HNA-3a plays an important role in the adhesion and activation of venous neutrophils under inflammation and specific shear (188), highlighting the potential of Slc44a2 as an adjuvant therapeutic target for DVT. Kindlin-3 is a key integrin activator that is expressed primarily in platelets and leukocytes (189), and deficiency of kindlin-3 in humans affects platelet aggregation and neutrophil adhesion. Kindlin-3-mediated integrin αIIbβ3 signaling in platelets is a prerequisite for the development of DVT. At the same time, kindlin-3 in platelets and neutrophils can influence DVT formation by coordinating the release of NETs (190). Lysophosphatidic acid (LPA) is a bioactive phospholipid released by activated platelets. LPA can stimulate the rapid release of NETs from neutrophils via a PAD4-dependent pathway, thereby promoting thrombotic stability. Therefore, LPA-NETs are potential targets for the prevention and treatment of DVT (191).

## 5 Treatment of DVT via NETs

Diagnostic and therapeutic strategies for DVT are hot topics of research nowadays. In clinical practice, the diagnosis of DVT requires a combination of clinical scores (e.g., Wells score), D-dimer testing, and imageological examination to evaluate the clinical predictive probability of DVT. If necessary, ultrasonography should be performed for suspected DVT, and CT imaging should be performed for suspected PE (192). Ultrasonography of leg veins and serial D-dimer assays were identified as having the potential to identify outpatients with a first episode of DVT (193). In addition, treatment of DVT is aimed at preventing thrombus spread, cardiopulmonary failure, death, and long-term complications. The mainstay of treatment for DVT is anticoagulation, and failure to initiate anticoagulation promptly and delays in anticoagulation may lead to disease progression (194). Thrombolytic therapy should be considered for patients with intermediate-high risk PE and clinical signs of hemodynamic decompensation (195). Also, the placement of permanent inferior vena cava filters may reduce the incidence of PE in patients at high risk for acute proximal DVT (196). In patients requiring DVT prophylaxis, pharmacologic interventions are recommended in the absence of contraindications to anticoagulants. Elastic compression stockings or intermittent pneumatic compressive devices may be considered when there are contraindications to anticoagulants (192, 197). Although the aforementioned diagnostic and therapeutic strategies for DVT have been clinically applied, limitations remain. For example, D-dimer diagnosis of DVT has limited sensitivity and specificity which is influenced by individual differences and disease factors. There is a risk of bleeding after both anticoagulation and thrombolysis. There is a lack of evidence on the safety of inferior vena cava filters. Therefore, the individualized prevention, diagnostic effectiveness, and therapeutic safety of DVT still need to be further explored, and the pathophysiological mechanism of DVT formation may provide us with new ideas.

NETs act on both the coagulation and immune systems during the occurrence of deep vein thrombosis. Platelets and coagulation factors, key components of thrombosis, induce and govern the formation of NETs. The interaction between NETs and immune cells causes inflammation and participates in the formation of DVT. Regarding the treatment of deep vein thrombosis, the following aspects can be considered (**Table 1**). Firstly, in terms of NETs themselves, DNA and histones are produced upon decondensation of neutrophil chromatin. To mitigate the effect of NETs on DVT formation, we can minimize the formation of DNA and histones or secrete enzymes that break down or regulate NETs, such as the inhibition of PAD4 (231). Secondly, the risk of DVT can be decreased by interfering with or inhibiting the formation of NETs by clearing or partially clearing neutrophils. Thirdly, there are specific NETs-binding proteins in the coagulation and immune systems, such as TF, IL-17, and IL-1β (238–240), which possess disease-related biological activities. If their action can be interfered with without affecting the body’s normal coagulation and immune capacity, they may potentially become more effective or safer antithrombotic agents (241–243). Therefore, there is an urgent need to better understand the function of NETs and the balance between their induction and inhibition to further tailor therapeutic approaches.

**Table 1:** Summary of DVT treatment strategies targeting NETs.

Strategies	Drugs	Outcome	Reference
cfDNA degradation	DNase I	Digestion of cfDNA in NETs, inhibition of coagulation, and effects on thrombolysis in a mouse model	(74, 126, 171, 173, 198–200)
Nucleosomal chromatin degradation	DNase Il3	Degradation of NETs to prevent DIC	(201, 202)
Histone inhibition	FSAP	Reduce histone toxicity to cells	(203)
	SPAs	Prevention of histone - mediated diseases associated with NETs	(204)
	Heparin	Form heparin-histone complexes, reduce endothelial damage and cytotoxicity,	(117, 205–207)
	PSA	Reduce histone and nets mediated cytotoxicity	(208, 209)
	Anti-H3Cit monoclonal antibody	Reduce inflammatory response and improve the survival rate of mice with sepsis	(210)
PAD4 inhibition	TcpC	Ubiquitination of PAD4 degrades, thereby inhibiting the formation of NETs	(211)
	F- and Cl-amidine	Reduce histone citrullination and inhibit the formation of NETs	(212, 213)
	GSK199 and GSK484	Selective inhibitors of PAD4 that inhibit NETs formation	(214)
ROS inhibition	Serum albumin	Scavenge mtROS and inhibit the formation and release of NETs	(215, 216)
	Erythropoietin‐induced hemoglobin subunit beta	Inhibit ROS and reduce the formation of NETs	(217)
	(+)-Borneol	Inhibition of PMA-induced NETosis	(218)
	Vitamin B1	Prevent excessive production of NETs from inflammatory diseases	(219)
Reduce NETs-platelets	APC	Reduce immune thrombosis	(220)
Charge-Charge Interactions	Lactoferrin	Inhibit the formation and spread of NETs	(221)
Modulate signaling pathway	EGCG	Regulation of STAT3/CXCL8 signaling pathway inhibits the formation of NETs, thereby suppressing the migration and invasion of colon cancer cells	(222)
	Glucocorticoid	Inhibition of LPS-induced NF-κB signaling pathway and formation of NETs	(223)
	Ginsenoside Rg5	Inhibition of ERK/NF-κb signaling pathway, neutrophil infiltration, NETs release, and DVT formation in mice	(224)
Inhibit the aggregation of neutrophils and macrophages	RvD4	Regulation of thrombotic disease, thereby improving thrombolysis	(225)
Caspase-1 inhibition	Caspase-1 inhibitor	Reduce the formation of thrombosis and the incidence of DVT in mice	(226–228)
IL-17A inhibition	IL-17A inhibitor	Inhibit platelet activation and the release of NETs, thereby inhibiting DVT	(229, 230)
IL-1β inhibition	CD39	Inhibit intravascular inflammation and thrombosis	(231–233)
vWF-FVIII Clearance	Stabilin-2	Reduce the release of NETs and DVT formation	(234)
Blood coagulation factor inhibition	Betrixaban	Prevention of venous thromboembolism in adults	(235)
	Dabigatran	Interference with the formation of NETs in mice models	(144, 201, 236)
NETosis inhibition	QJHTD	Inhibit the production of NETs and regulate inflammatory responses	(237)

### 5.1 Inhibit the formation of NETs

#### 5.1.1 DNase I therapy

Deoxyribonuclease (DNase), a nucleic acid endonuclease, can digest single- or double-stranded DNAs. The use of DNase I reduced thrombosis in different mouse models of DVT (126, 171, 173, 198). cfDNA is a biomarker for NETs, and cfDNA levels may be a valuable asset to assist in the diagnosis of DVT (74). An earlier study established that cfDNA levels in elderly patients with venous thromboembolism might reflect the degree of inflammation and may be used as a biomarker for risk of death stratification in this population (244). It has been observed that DNase I is more effective in reducing thrombus size than in depleting neutrophils because it breaks down not only NETs but also cfDNA (198). Regarding cfDNA-rich thrombi, NETs may increase the ability of the thrombus to resist cleavage (245), suggesting the need for thrombolytic strategies that target the DNA component. To maintain the stability of DNase I and to protect the efficient transport of DNase I, nanoparticles encapsulated with DNase I have been used. This long-lasting DNase I improves the stability and half-life of the enzyme and inhibits NF-κB activation and cytokine levels, thereby suppressing neutrophil and NETs formation (246). 2C5 is a monoclonal antibody that is highly specific for intact nuclear histones and can target binding to NETs (247). Targeted nanocarriers of 2C5 and DNase I can target NETs and have clinical implications for the targeted removal of NETs (248).

DNase I may inhibit coagulation and impact thrombolysis by catabolizing cfDNA. As observed in patients with deep vein thrombosis (74), cfDNA fragments enhanced the intrinsic coagulation pathway (199), leading to tissue hypoxia and endothelial damage. The results of a cross-sectional study demonstrated that DNase I may limit thrombin production by hydrolyzing cfDNA and preventing venous thrombosis during aging (200). In mouse models, DNase I treatment significantly reduced cfDNA, immune cell infiltration, and thrombin-antithrombin-iii production. Notably, medium-term use of DNase I prevented venous thrombosis in tumor-bearing mice without significant hemorrhagic effects (249). An earlier study evinced that the combination of DNase I and t-PA accelerated *in vivo* thrombolysis during the early phase of acute ischaemic stroke (152). Increasing evidence of the benefits of DNase I application in different disease models. From the degradation of cfDNA to inhibition of NET formation, DNase I therapy is anticipated to be a novel therapeutic concept for the treatment and prevention of thrombosis.

DNase I-like 3 (DNase Il3), also known as DNase γ, is mainly secreted by macrophages (250) and targets DNA-protein complexes such as nucleosomes (201). Both DNase I and DNase Il3 degrade NETs in blood circulation. They differ in that DNase Il3 degrades the chromatin of nucleosomes on its own and is inhibited by fibrinolytic enzymes. In contrast, DNase I only binds to proteases (e.g., fibrinolytic enzymes) to degrade chromatin (251). Defective DNase Il3 expression in diabetic hepatocellular carcinoma (HCC) tissues is a key cause of impaired DNA degradation in NETs, possibly through upregulation of the cyclic GMP-AMP synthase (cGAS) and NF -κB signaling pathway (202). Meanwhile, DNase Il3 and DNase I may prevent disseminated intravascular coagulation (DIC) by degrading NETs (201). At present, we only know that DNase Il3 and DNase I similarly degrade NETs, but the exact mechanism in DVT needs to be explored.

#### 5.1.2 Histone inhibition

Histones are components of NETs, and high levels of H3Cit are associated with venous thrombosis (252). Factor VII activating protease (FSAP) can be activated *in vivo* by the release of histones from NETs, and this activation may reduce the toxicity of histones on cells (203). Meanwhile, small polyanions (SPAs) were found to be effective in inhibiting histone and platelet activation and were able to prevent histone-mediated diseases associated with NETs, particularly sepsis and DVT. Therefore, a non-toxic SPA may be able to inhibit NETs and limit histone-mediated pathological responses and has already been identified for clinical development (204). Some studies have validated that effective blockade of circulating H3Cit may be a potential therapeutic approach for the treatment of endotoxemia (210), and we hypothesize that this approach can also be applied to the treatment of DVT.

In addition, some anti-histone agents can also target inactivated histones. In addition to its anticoagulant effects, heparin can bind cell surface receptors or macromolecules, including P-selectin and intercellular adhesion molecule-1, and inhibit interactions between endothelial cells and blood cells (e.g., leukocytes, platelets), with clinical value in thromboprophylaxis (253). In sepsis, heparin binds positively charged histones through high-affinity electrostatic interactions, allowing the formation of heparin-histone complexes without cytotoxicity (205). Heparin breaks down NETs and prevents histidine-induced platelet aggregation (117). Anti-thrombin affinity depleted heparin (AADH) binds directly to histones and can effectively block histone-mediated cytotoxicity (206). In an LPS-induced mouse model, optimized Chondroitin sulfate E 19-mer binds tightly to histone H3, and this Heparinoid reduces endothelial cell damage by histone H3 (207). Ploysialic acid (PSA) consists of α-2,8-linked N-acetylneuraminic acid residues. PSA significantly reduces histone and NETs-mediated cytotoxicity through electrostatic interactions with histones (208, 209). Anti-H3Cit monoclonal antibodies have been investigated which neutralize H3Cit to attenuate the inflammatory response and thus improve survival in septicemic mice (210). Overall, the immunological role of histones in mediating endothelial injury and activating the coagulation system is not fully understood as inhibition of histones in DVT formation has been little studied.

#### 5.1.3 PAD4 inhibition

PAD4 is mainly expressed in neutrophils and it catalyzes the conversion of histone arginine to citrullination. In the DVT mouse model, endothelial activation and platelet function were stimulated by LPS but not by PAD4 deficiency, and PAD4 ^-/-^ mice formed NETs-free thrombi. However, after infusion of neutrophils from wild-type mice, PAD4 ^-/-^ mice formed NETs-containing thrombi. This suggests that venous thrombosis is dependent on PAD4 in neutrophils and that neutrophil activation and PAD4 could be potential drug targets for deep vein thrombosis (254). TcpC is a virulence factor for uropathogenic E. coli. TcpC is a PAD4-targeting E3 ubiquitin ligase that promotes the degradation of PAD4 via the ubiquitin-proteasome pathway. TcpC can promote the ubiquitinated degradation of PAD4 to inhibit the formation of NETs, suggesting a novel mechanism for TcpC-mediated bacterial immune evasion (211).

Although several PAD inhibitors have been identified (e.g., paclitaxel, minocycline, and streptomycin) (255), these drugs are reversible and non-PAD4 specific (256) and it is not known whether they inhibit DVT formation. In addition, irreversible inhibitors of PAD4 (e.g., F- and Cl-amidine) covalently bind to Cys645 at the PAD4 active site (212), significantly reducing histone citrullination and inhibiting the formation of NETs (213). Through DNA coding library screening and compound optimization, GSK199 and GSK484 were developed as potent selective inhibitors of PAD4 that inhibit the formation of NETs (214). To date, no PAD4 inhibitors have been available for DVT treatment. However, these PAD4 inhibitors should be a high affinity and highly specific to be used successfully in DVT patients. This may be the focus of future breakthroughs needed.

#### 5.1.4 ROS inhibition

The production of ROS has been described as an important signal for the formation of NETs, so it is important to balance the production and removal of ROS. Since the Raf-MEK-ERK pathway activates NADPH oxidase to produce ROS, the formation of NETs can be inhibited by blocking the Raf-MEK-ERK pathway, making this pathway a potential target for NET inhibitors (60). Mitochondrial ROS (mtROS) are generated by the NADPH oxidase non-dependent pathway, and upregulation of mtROS promotes the release of NETs. A degree of spontaneous NETs formation has been shown to occur in serum-free cultures (257). Serum albumin exhibits mtROS scavenging activity, thereby inhibiting the formation and release of NETs (215, 216). In a case report of a critical patient with COVID-19, it was noted that Erythropoietin-induced hemoglobin subunit beta may inhibit ROS and reduce NETs formation (217). (+)-Borneol reduces ROS levels in activated neutrophils and inhibits NETosis triggered by PMA stimulation *in vitro* (218). The antioxidant properties of vitamin B1 could have an effect on ROS during NETosis (219). The above-mentioned are *in vitro* studies, and we need more detailed and comprehensive information about the mode of action and protective properties of the drugs.

#### 5.1.5 Other NETs inhibition methods

Some other methods of NETs inhibition are listed below. Endothelial cells expressing Thrombomodulin (TM) can maintain vascular homeostasis. One of these, Recombinant Thrombomodulin (rTM), binds competitively to Mac-1, interferes with ANCA binding to neutrophils, and inhibits ANCA-induced NETs formation, helping to resolve intravascular inflammation and immunothrombosis (258). Activated protein C (APC) is a multifunctional serine protease that protects barrier function and reduces vascular permeability. Recent evidence suggests that in non-human primate models, APC reduces immunothrombosis by inhibiting NETs formation and reducing NETs-platelet binding (220). Lactoferrin, an endogenous inhibitor of NETs, may inhibit NET formation by blocking the propagation of NETs through charge-charge interactions (221). Some components of herbs or foods (e.g., Dihydrotanshinone I (DHT) and Kaempferol (Kaem)) can reduce H3Cit expression and release of NETs (259, 260). Some drugs can inhibit the formation of NETs by modulating signaling pathways. It has been shown that epigallocatechin-3-gallate (EGCG) inhibits the formation of NETs through modulation of the STAT3/CXCL8 signaling pathway, thereby inhibiting the migration and invasion of colon cancer cells (222). Glucocorticoids are anti-inflammatory agents that inhibit LPS-induced NF-κB signaling, thereby suppressing neutrophil activation and reducing the formation of NETs (223). Ginsenoside Rg5, a natural saponin in P. ginseng and P. notoginseng, interacts with the P2RY12 variant to decrease the inflammatory response by inhibiting the ERK/NF-κb signaling pathway, suppressing neutrophil infiltration and NET release, and interfering with DVT formation in mice. This was accompanied by a reduction in plasma IL-6, IL-1β, and TNF-α release, which may have clinical implications for the prevention of DVT-related clinical disorders (224). These findings provide new research directions for clinical antithrombotic therapy.

### 5.2 Inhibit inflammatory response

Immune cells play a crucial role in the inflammatory response. In other words, neutrophils, monocytes, macrophages, endothelial cells, and platelets interact with each other and play a key role in the occurrence of venous thrombosis. After the onset of ischemia, both neutrophil and monocyte count rapidly increase (261). It has been shown that CD11b^+^Ly6C^Hi^ monocytes do not significantly affect venous thrombosis, but their terminal effector cells CD11b^+^Ly6C^Lo^ monocytes/macrophages, are essential for venous thrombosis, possibly mediated through the activity of IFN-γ-directed lysis proteins (262). Bertin reported that a decrease in NK cells led to a decrease in NET and thrombus formation and that the production of NETs was dependent on the secretion of IFN-γ by NK cells, illustrating that NK cells are required for thrombus formation (263). NETs were found to shift macrophage polarization towards a reparative phenotype *in vitro*, thereby displaying anti-inflammatory properties (264). Remarkably, NET proteins persist long after DNA degradation, possibly attributed to the fact that DNase I promotes the uptake of NETs by macrophages *in vitro* (265). Resolvin D4 (RvD4), an endogenously biosynthesized specialized pro-resolving mediator (SPM), inhibits NETosis by impeding the aggregation of neutrophils and macrophages in thrombi, modulating the severity of thrombo-inflammatory disease *in vivo* and thus improving thrombolysis (225).

NETs and inflammatory vesicles can synergistically promote venous thrombosis. NLRP3 is a molecular complex that primarily converts caspase-1 and caspase-11 into their active forms, leading to cleavage and activation of IL-1β and IL-18 (266), with IL-1β regulating neutrophil recruitment and activation. Stimulation of neutrophils was found to induce both NET and caspase-1 activation, while inhibition of caspase-1 lowered the incidence of DVT, and lastly, selective caspase-1 inhibitors reduced the formation of thrombi in mice (226). Drugs that inhibit caspase-1 activity are also currently in development (227, 228). In the stenosis model of DVT, NLRP3 deficiency leads to a reduction in the density of NETs in the thrombus, while NLRP3 inhibitors will also reduce the toxic effects of NETs (267). The above results provide new ideas for the prevention and treatment of DVT.

### 5.3 Affect coagulation system

Deep vein thrombosis occurs at the crossroad of inflammation and coagulation dysregulation. Interleukin 17A (IL-17A) is a pro-inflammatory cytokine. Studies have shown that NETs provide carriers for IL-17A in non-alcoholic steatohepatitis and SLE, and that IL-17A may be potentially involved in inflammatory and fibrotic processes (240, 268). Furthermore, IL-17 in combination with TNFα has procoagulant and prothrombotic effects on blood vessels (269, 270). Platelets can express the IL-17A receptor (IL-17RA) (271). Earlier studies have described that IL-17A induces platelet aggregation and activation, regulates the release of NETs to enhance neutrophil infiltration, and promotes deep vein thrombosis (229). As anticipated, IL-17A monotherapy was found to inhibit platelet activation and neutrophil activity in a mouse model (230). Therefore, this may be a new target for the clinical treatment of DVT. Ectonucleotidase tri(di)phosphohydrolase-1 (ENTPD-1, also known as CD39) is an extravascular enzyme located on the surface of neutrophils and vascular endothelium that inhibits intravascular inflammation and thrombosis by hydrolyzing the phosphodiester bond of nucleotides released from activated cells. IL-1β, a key promoter of venous thrombotic inflammation, is inhibited by CD39, thus inhibiting the link between inflammation and coagulation *in vivo*; this is an important direction in the fight against venous thrombosis (231–233).

In addition, the effects of thrombin, fibrin, and fibrinolysis on shear-induced NETosis have been investigated. They found that fibrin suppresses shear-induced NETosis. They believe this suppression is beneficial because it protects neutrophils from large interstitial shear stresses within occlusive thrombi (272). Stabilin-2 is an endocytic scavenger receptor that mediates the clearance of the von Willebrand factor-VIII complex (FVIII) and reduces the release of NETs and DVT formation (234). The incidence of DVT in FXI-deficient patients is relatively low, and research on FXI therapies regarding FXI is ongoing (200). In addition, one study reported that betrixaban, an orally-administered factor Xa inhibitor, has been approved for the prevention of venous thromboembolism in adults (235). NETs formation by neutrophils can be interfered with by treating mice with the factor IIa inhibitor dabigatran (201, 236).

### 5.4 Other related prevention and therapy

Concerning treatment, in addition to catabolism of NETs and inhibition of NET-mediated inflammation and coagulation, perhaps autophagy (273), complementation, miRNA modulation, and herbal prescriptions could also be considered. Experimental results of PMA-induced NET production by neutrophils have shown that Qing-Jin-Hua-Tan-Decoction (QJHTD), a classic famous Chinese ancient prescription, can inhibit the production of NETs and also modulate bacterial and inflammatory responses (237). In addition, certain factors influencing the formation of NETs in other diseases might also be a new direction for DVT prevention and control. It has been observed that cancer patients have significantly down-regulated miRNAs before venous thrombosis (274), which may be diagnostic. Exogenous hydrogen sulfide (H2S) was able to reverse the expression levels of Bax, Bcl-2, phosphorylated p38 MAPK, and P-selectin and significantly inhibited homocysteine-induced cellular ROS production, platelet activation, and NET formation, thereby protecting endothelial cells (275). Furthermore, NETs play a leading role in thrombosis in anti-phospholipid syndrome (APS). Defibrotide is a heterogeneous mixture of polyanionic oligonucleotides that counteracted neutrophil-mediated thrombotic inflammation in APS in a mouse model (276). In short, treatment strategies for other diseases may potentially apply to the management of DVT as well.

## 6 Conclusions and perspectives

Inflammation and coagulation are two crucial factors affecting DVT formation, with NETs linking the two and occupying an essential position. From the standpoint of NET-mediated DVT formation, NETs are potential therapeutic targets for the prevention and treatment of patients with DVT. Although some evidence has suggested mechanisms related to the formation of DVT and NETs, the pathogenesis of DVT is not well understood. Furthermore, the immunological and pathological role of NETs in DVT needs further investigation. It has been noted in the study that serum surrogate markers of NETosis appear to predict necrotizing small bowel colitis in neonatal mice (277) and that NETs may be a practical predictor of portal vein thrombosis in liver cirrhosis (278). Even so, it has been proposed that NETs deposited in diseased organs are degraded at any time, and the quantification of NETs in plasma may not be accurate (279). Therefore, the assessment and quantification of NETs are particularly essential in the diagnosis of DVT. In the future, we need to establish predictive indicators of DVT associated with NETs, especially the establishment of sensitivity and specificity indicators. Similarly, targeted therapies for NETs (e.g., DNase I therapy, histone inhibitor, PAD4 inhibitor, etc.) continue to require thorough investigation and analysis in preclinical and clinical studies, regardless of whether they degrade NETs or inhibit their formation. It is well known that DNase I treatment may reduce extracellular genomic DNA, but it does not eliminate extracellular histones, which are known to promote thrombosis (280). This led us to conclude that a combination treatment strategy may be superior to DNase I monotherapy. Meanwhile, DNase I can be inhibited by actin, whereas DNase Il3 can be inactivated by heparin and cleaved by fibrin (251). Thus, the duration of DNase I activity still requires further *in vivo* experimental studies. In the long run, in addition to evaluating the safety and efficacy of drugs, there is a greater need to improve the specificity and half-life of drugs, for example, by developing targeted drugs against histones, PAD4, and ROS.

The formation of neutrophil-stimulated NETs is itself the first line of defense against a wide range of pathogens, and we need to consider whether a reduction in NETs would affect the body’s immune function, e.g., whether the risk of infection would increase in critically ill patients. Therefore, it is essential to remove the harmful products released by NETs and prevent over-activation of the immune system without destroying the normal function of neutrophils and NETs. Although clinically well-established antithrombotic strategies are available, they inevitably increase the risk of bleeding. Therefore, by studying the inflammatory mechanisms of DVT formation, it may be possible to find strategies to replace or synergize conventional therapies. In conclusion, to elucidate these questions, the role of NETs in deep vein thrombosis should be clarified, and further research is warranted.

## Author contributions

JWM contributed to conception and design of the review. MY wrote the first draft of the manuscript. MY, JCM, DW, CF, ZW and TG contributed to the editing and revising of this review. All authors contributed to manuscript revision, read, and approved the submitted version.

## Glossary

**Table:**

AADH	anti-thrombin affinity depleted heparin
ADP	adenosine diphosphate
aggNETs	aggregated NETs
APC	activated protein C
APS	antiphospholipid syndrome
cfDNA	cell-free DNA
CG	cathepsin G
CGD	chronic granulomatous disease
COVID-19	Coronavirus disease 2019
CTEPH	chronic thromboembolic pulmonary hypertension
CXCR2	C-X-C motif chemokine receptor 2
DAMPs	damage-associated molecular patterns
DHT	Dihydrotanshinone I
DNase	Deoxyribonuclease
DNase Il3	DNase I-like 3
DVT	deep venous thrombosis
ENTPD-1	Ectonucleotidase tri(di)phosphohydrolase-1
ERK	extracellular signal-regulated kinase
FSAP	Factor VII activating protease
G-CSF	granulocyte colony-stimulating factor
GPIbα	glycoprotein Ibα
GSH	glutathione
H3Cit	Histone H3 Citrullination
HIF-1α	Hypoxia-inducible factor 1-alpha
HMGB1	high mobility group protein B1
HNA	human neutrophil antigen
HPAEC	human pulmonary artery endothelial cells
ICAM-1	intracellular adhesion molecule 1
IL	interleukin
kaem	Kaempferol
LPA	Lysophosphatidic acid
LPS	lipopolysaccharides
MEK	mitogen-activated protein kinase
MLCK	myosin light chain kinase
MMP	matrix metalloprotease
MPO	myeloperoxidase
MSU	monosodium urate
mtDNA	mitochondrial DNA
mtROS	mitochondrial ROS
NADPH oxidase	nicotinamide adenine dinucleotide phosphate oxidase
NE	neutrophil elastase
NETs	neutrophil extracellular traps
NLRP3	Nod-like receptor protein-3
oxLDL	low-density lipoprotein
P2RY_12_	purinergic receptor type Y, subtype 12
PAD4	protein-arginine deiminase type 4
PDI	protein disulfide isomerase
PE	pulmonary embolism
PKC	protein kinase C
PMA	phorbol-12-myristate-13-acetate
PNAs	platelet-neutrophil aggregates
olyp	inorganic polyphosphate
PSA	Ploysialic acid
PSGL-1	P-selectin glycoprotein ligand -1
PTS	post-thrombotic syndrome
QJHTD	Qing-Jin-Hua-Tan-Decoction
Raf	rapidly accelerated fibrosarcoma
RAGE	receptor of advanced glycation end
ROS	reactive oxygen species
RvD4	Resolvin D4
SLE	systemic lupus erythematosus
SPAs	Small polyanions
SPMs	specialized proresolving mediators
TF	tissue factor
TFPI	tissue factor pathway inhibitor
TM	Thrombomodulin
TNF-α	tumor necrosis factor-α
TXA2	thromboxane A2
VCAM-1	vascular cell adhesion molecule 1
VTE	venous thromboembolism
vWF	von Willebrand factor.
